# Incidence and clinical profile of herpes zoster in primary care in Bahrain – A cross-sectional study

**DOI:** 10.5339/qmj.2025.72

**Published:** 2025-08-22

**Authors:** Zahra Zabar, Zahra Ayoob, Huda Malalla, Maryam Jadeed, Afaf Merza, Adel AlSayyad

**Affiliations:** 1 Primary Healthcare Centers, Manama, Bahrain; 2 Ministry of Health, Manama, Bahrain; 3 Epidemiology & Public Health, Ministry of Health, Manama, Bahrain; 4 Department of Family and Community Medicine, College of Medicine and Medical Sciences, Arabian Gulf University, Manama, Bahrain *Email: mariam.jadeed@gmail.com

**Keywords:** Bahrain, herpes zoster, varicella, primary health, profile

## Abstract

**Background:**

Herpes zoster (HZ) is a secondary viral infection that results from the reactivation of latent varicella zoster virus, characterized by dermatological manifestations and neurological sequelae. The incidence of HZ increases with age and is higher among immunocompromised individuals. While the global literature extensively documents HZ disease and its impact, there is a paucity of data in regional studies. Despite the availability of vaccines, HZ poses a public health challenge, especially in regions with limited healthcare access, underscoring the need for better surveillance and management strategies globally, regionally, and nationally.

The study aims to estimate the incidence of HZ among attendees of primary healthcare facilities in the Kingdom of Bahrain, analyze the demographic distribution of patients based on age, sex, and risk factors, and gain insights into the clinical presentation and the most common complications within the local society.

**Methods:**

This study used a retrospective cross-sectional design, targeting all patients who visited governmental healthcare facilities and were reported to have been diagnosed with HZ in 2021, according to electronic medical records. Patients were contacted via phone to collect specific information related to the episodes they experienced, while additional information was retrieved from electronic health records (EHR). Informed consent was obtained from all participants. During the calls, five patients declined to provide details about the episodes; their decision was respected, and only the information available in their EHR was used. All collected data were systematically recorded in an Excel spreadsheet for analysis.

**Results:**

The total incidence of HZ was 59.09 per 100,000 population. The median age was 42.8 ±19 years, with a higher prevalence observed in males (53.4%). Of the study participants 79.1% were Bahrainis, 22.3% had diabetes, and 3% had other comorbidities. The most frequently reported clinical manifestations were rash (79.9%) and pain (15.8%). The trunk (30.5%), back (19.5%), and abdomen (13.9%) were the most commonly reported locations affected by HZ. Antiviral treatment was administered to 65.2% of the patients. The most commonly reported complications included post-herpetic neuralgia (6.7%) and cellulitis (4.4%).

**Conclusion:**

Individuals in older age groups exhibit a significantly higher likelihood of developing HZ infection along with the associated post-infection complications. This finding aligns with those from other studies. It is recommended to implement interventions aimed at reducing both the incidence and morbidity of HZ, particularly targeting those at higher risk.

## 1. INTRODUCTION

Herpes zoster (HZ) is a secondary disease that results from the reactivation of the latent varicella zoster (VZ) virus that remains dormant in the sensory ganglia.^1^ The disease is marked by an eruption of a characteristic skin rash that is confined to one or two adjacent dermatomal distributions, not crossing the midline, and usually appears on the face or trunk.^1^ HZ can occur at any age, but the highest burden of the disease is observed in older adults.^1^

Studies show that the incidence rate significantly increases with age, with approximately 50% of individuals who live beyond 85 years experiencing an episode of HZ.^2^ In addition to age, multiple risk factors have been identified in the literature, including immunosuppression, ethnicity, family history, physical injury, and other comorbidities.^3^ Several studies have concluded that female gender is an independent risk factor for HZ, even after controlling for age.^4^ There has been an increasing attention towards HZ infection in recent years. The incidence rate in Western countries is estimated to be 3.6 per 1,000 in the United States and 5.23 per 1,000 in the United Kingdom.

A systematic review that analyzed publications from China, Taiwan, and Hong Kong found an annual incidence rate ranging from 2.9 to 5.8 per 1,000 person-years in individuals aged 50 years and above.^5^ Similarly, a study from Australia estimated the incidence rate for individuals aged 50 years and above to be 10 per 1,000.^6^ In comparison, studies conducted in the Middle East showed an incidence rate of 4.5 per 1,000 adults in Israel^7^ and 2.63 per 1,000 adults in Turkey.^8^

Regionally, there was a paucity of published evidence. A recent study conducted in Qatar reported an increase in the incidence rate from 0.98 per 1,000 to 3.62 per 1,000 between 2012 and 2017, respectively. Another single-center study from the Kingdom of Saudi Arabia showed an incidencerate of 6.3 per 1,000 among attendees at dermatology clinics.^9,10^

Several articles discuss the complications of HZ, with post-herpetic neuralgia (PHN) being the most commonly mentioned complication due to its complex treatment that may persist for several months to years.^1^

In addition to neurological sequelae, complications can vary from post-inflammatory hyperpigmentation and scarring to more sever cardiac, ophthalmic, and gastrointestinal conditions.^4^

Furthermore, HZ and its associated complications have an economic impact that cannot be overlooked, including loss of work hours, absenteeism, a frequent need for home care, and the chronic nature of symptoms, all of which contribute to the strain on an already overburdened global healthcare system.^11^

Considering the complications and the burden of the disease on both the health and economy, coupled with the understanding that antiviral treatment is only effective and successful when commenced early in the course of the disease, this has encouraged the development of prevention strategies in the form of the HZ vaccine.

The first attempt to prevent HZ through immunization was undertaken by the Food and Drug Administration, which introduced and approved different types of vaccines that can be administered at different age groups.^12^ Numerous studies have concluded that the introduction of the HZ vaccine reduces the burden of illness and its associated complications.^12–14^

To the best of our knowledge, this is the first study in Bahrain that addresses the incidence, clinical characteristics, and demographic distribution of HZ.

The aim of this study was to address the existing gap in the literature by providing a comprehensive description of HZ infection in the Kingdom of Bahrain. The objectives of the study include estimating the incidence of HZ among attendees of primary healthcare facilities, analyzing the demographic distribution of patients based on age, sex, and risk factors, as well as understanding the clinical presentation and the most common complications within the local population.

## 2. METHODS

This retrospective cross-sectional study was conducted in the primary healthcare setting in the Kingdom of Bahrain. All cases reported as HZ through the electronic medical record system in the year 2021 were included as study subjects. The criteria for exclusion involved cases where the information in the medical records did not correspond to that of an HZ case or if the patient stated that a specialist had provided a different diagnosis. Data was collected by obtaining access to the infectious disease report through the electronic medical record system known as “I-Seha”. Data was extracted from this report, and medical records were reviewed. Individuals reported in the year 2021 were contacted via the phone to collect further information regarding their clinical presentation and medical history. The researchers’ access to the electronic health record data of patients did not raise any ethical concerns, as this was clearly stated in the research proposal, which received approval from the research and health committee. Patients were contacted to obtain further information, particularly details that may not have been documented by the physician in the system. For individuals who opted not to provide additional information, no further attempts were made to involve them, and only the data already available was used.

Demographic information was collected for all cases, including age, sex, nationality, and relevant medical history. Additionally, data on clinical presentation, antiviral use, and the presence and type of complications were collected. The data was analyzed using both Microsoft Excel and the Statistical Package for the Social Sciences software version 23.0 (SPSS, IBM Corporation, NY, USA). Descriptive statistics were calculated to analyze sociodemographic data, such as sex, nationality, and comorbidities, which are presented as frequencies and percentages. Numerical variables are presented as mean and standard deviation (SD). Age-specific incidence was estimated using national population data as the denominator. Binary logistic regression was used to evaluate independent risk factors associated with the development of complications. A p value of less than 0.05 was considered statistically significant.

Ethical approval was obtained from the research committee in primary care in Bahrain before conducting the research (PHC-IRB-2022-04-E).

## 3. RESULTS

The total number of patients who developed HZ infection in 2021 was 882, with 684 (77.6%) responding to phone calls, and five of those who answered refused to participate. Regarding gender distribution, 471 (53.4 %) of the participants were male and 411 (46.6%) were female. The median age of the study subjects was 42.8 ± 19 years, with the largest proportion of the sample being patients aged between 50 and 59 years. A total of 698 patients were Bahraini (79.1%) compared to 184 (20.9%) who were non-Bahraini.

**Figure 1:**
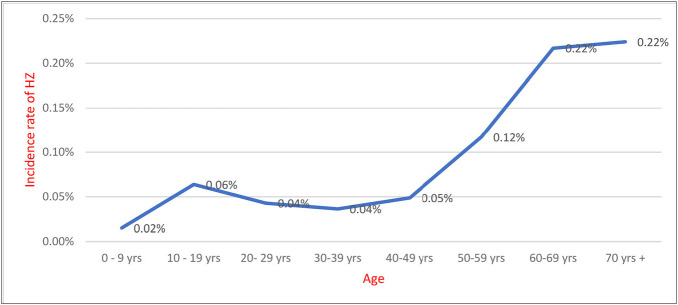
Age-specific incidence of HZ in Bahrain for the year 2021.

The total incidence rate of HZ in 2021 was 59.09 per 100,000 population. In general, the age-specific incidence tends to increase with age, with a steep rise after the age of 50 years. Interestingly, among patients less than 50 years of age, the age group of 10–19 years exhibited a higher incidence, reaching 63.82 per 100,000 population (Figure 1).

Regarding the associated clinical conditions, 196 (22.3%) patients were diabetics, while five (0.6%) patients had previously undergone organ transplantation. Moreover, 21 (2.4%) were diagnosed with an autoimmune disease, including SLE (systemic lupus erythematosus), RA (rheumatoid arthritis), psoriasis, and IBD (inflammatory bowel disease). Furthermore, 17 (1.9%) patients with HZ had a form of cancer (Table 1).

The most predominant symptom reported was a rash, which was the chief complaint in 705 (79.9%) patients, followed by pain in 139 (15.8%) patients and itchiness in 21 (2.4%) patients. Concerning the locations affected, the trunk, back, and abdomen were the most frequently involved areas, with respective percentages of 30.5%, 19.5%, and 13.9%. Additionally, 100 (12.9%) patients had facial involvement. The right side of the body was affected in 356 (54.1%) patients, while the rash was present on the left side in 294 (44.7%) patients. However, 8 (1.2%) participants had a rash that extended both sides (Table 2).

**Figure 2:**
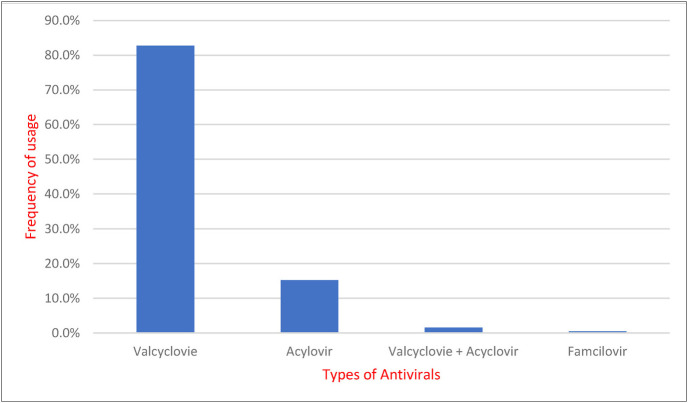
Type of antivirals used in patients requiring antiviral treatments (*N* = 466).

A total of 466 (65.2%) patients with HZ were prescribed an oral antiviral agent, which included either valcyclovir (82.7%) or acyclovir (15.2%), with 1.6% requiring both treatments. Only 0.4% of the cohort received famiclovir. Among the participants, eight (1.3%) patients required admission, specifically those with severe disease or significant comorbidities (Figure 2).

Among those who responded to phone calls, 105 (15.4%) patients reported experiencing complications related to the HZ virus. The study shows that PHN is the most prevalent complication, affecting 46 (6.7%) patients, followed by cellulitis, which occurred at a rate of 4.4%. Additionally, six (0.9%) patients had facial palsy, while 13 (1.9%) patients experienced a recurrence of the HZ episode (Tables 2 and 3).

Binary logistic regression was conducted to assess the factors associated with the occurrence of complications after HZ infection. The results indicate that older age statistically significantly (p < 0.005) increases the likelihood of developing post-infection complications, as shown in Table 4. The use of antivirals seems to play a statistically significant role (p < 0.005) in increasing HZ virus-related complications by a factor of 2 (95% CI 1.15–3.13, p = 0.008). Conversely, other factors such as sex, the presence of diabetes, cancer, autoimmune diseases, or undergoing organ transplantation were not found to be statistically significantly associated with the complications of the disease (Table 4).

## 4. DISCUSSION

The baseline data represents the characteristics of patients with HZ in Bahrain. Existing literature on the predominance of HZ between males and females presents varied and inconclusive findings.^10,13–15^ Our analysis, which accounts for the gender composition of the country, reveals a higher incidence of HZ among females. The calculated incidence rates were 71 per 100,000 females and 50.9 per 100,000 males. This discrepancy can be attributed to the different settings of each study, the varying research methods, and the diverse surveillance and reporting techniques across different countries.

Approximately 22% of patients had diabetes, a finding that aligns with the results from other studies. Vukelić et al. found that the majority of patients suffered from comorbidities, with approximately 14% having diabetes and 13% having malignancies.^16^ Similar findings have emerged from regional studies; for example, a study conducted in Saudi Arabia identified diabetes (21.5%) and immunocompromised conditions (19.4%) as the most common diseases in patients with HZ.^17^

The predominant complaints among patients were primarily rash (79.9%), followed by pain (15.8%) and itchiness (2.4%). In contrast, differences were observed in other countries such as India, Iran, and Malaysia, where pain was the most common presenting complaint, followed by itchiness.^13,18,19^ Our data also showed that the trunk was the most commonly involved location in clinical presentations, followed by the back, abdomen, and face, respectively. This pattern was observed in other studies, which similarly found the thoracic segment to be the most commonly affected.^13,18,20^

Regarding complications, PHN was identified as the most common. Numerous other studies have reported similar findings both at regional and international levels.^3,17,18^

Additionally, other studies have explored the financial consequences of HZ on the healthcare system. The estimated duration of hospital stay associated with this condition was 7.9 days, with an additional 14.6 days dedicated to rehabilitation. The duration of visits to the emergency department ranged from 5.8 to 14.2 hours.^6^

In terms of medical visits, HZ involved 2.4 consultations with general practitioners, and 1.77% of patients with HZ were referred to specialists, with a total cost of 181,000 US dollars per patient annually. Antiviral treatment was administered to 73.5% of patients, amounting to approximately 8.2 million US dollars. This highlights the persistent economic burden on the healthcare system.^6^

Moreover, unlike similar studies conducted in the region, more than 80% of the antiviral prescribed in our cohort was valcyclovir, followed by acyclovir and famciclovir. In Saudi Arabia, acyclovir was the most commonly prescribed medication.^17^ A different trend was observed in China, where valaciclovir and famciclovir were the most commonly prescribed antivirals.^21^ Despite this observed difference, studies did not indicate any superiority in favor of a particular medication,^22,23^ suggesting that the choice of an antiviral may depend on factors such as its availability, the preferences of patients or physicians, and the cost of the medication.

Except for older age and antiviral use, we did not find any significant association between disease complications and other risk factors. Although the early administration of antivirals has been shown to reduce the risk of complications as reported in the literature,^24^ our conflicting data may be explained by the late presentation of patients with HZ to healthcare facilities, resulting in a late initiation of antivirals, which was not assessed in this study. It is important to note that the severity of the disease at the time of presentation was not assessed, and this factor could also contribute to this outcome.

In comparison to previous studies, there is an association between age and the increasing incidence of zoster.^7,25^ Our results indicate that the incidence increases after the age of 50 years, which various studies have linked to a gradual decline in cell-mediated immunity. Furthermore, we observed a different trend showing a peak in HZ cases among individuals aged 10–19 years. This phenomenon was also noted in Germany, where an increase in incidence followed the introduction of the VZ vaccine in childhood, leading to the hypothesis of exogenous boosting.^20^

## 5. STRENGTHS AND LIMITATIONS

This study has several strengths. Importantly, it is the first national study to assess the clinical characteristics and trends of the HZ virus in Bahrain, using a sample that represents patients from all public healthcare facilities in the country.

However, certain limitations must be considered. The data excludes patients from private sector and military hospitals, potentially affecting the true incidence rate of the disease. Additionally, some data was collected through patient interviews conducted via phone, which introduces the risk of recall bias.

## 6. CONCLUSION

The likelihood of developing HZ infection and its associated post-infection complications significantly increases with older age groups. It is recommended to implement interventions aimed at reducing incidence and morbidity that specifically target higher risk groups. In the context of Bahrain, future plans should include conducting a comprehensive study on the financial burden of HZ, assessing both direct medical costs and indirect costs related to productivity loss and long-term disability. This approach will provide valuable insights into the economic impact of HZ and help prioritize healthcare resources effectively.

Moreover, enhanced public and healthcare provider education on the benefits of vaccination and early treatment options for HZ and its complications are crucial. Establishing a national registry for HZ cases in Bahrain could facilitate ongoing surveillance, improve patient outcomes through timely interventions, and inform future public health strategies.

## LIST OF ABBREVIATIONS

**Table:** 

CI	Confidence Interval
HZ	Herpes Zoster
IBD	Inflammatory Bowel Disease
I-Seha	Electronic Medical Record System
PHN	Post-Herpetic Neuralgia
RA	Rheumatoid Arthritis
SD	Standard Deviation
SLE	Systemic Lupus Erythematosus
SPSS	Statistical Package for the Social Sciences
VZ	Varicella Zoster

## ETHICAL CONSIDERATIONS

The study was approved by the primary healthcare research committee. Before data collection, consent from participants was obtained through phone calls. To ensure patient confidentiality, a serial number was assigned to each participant, and all data were coded during the analysis.

The ethical committee is a part of research committee in Bahrain. Concurrently, our study is ethically approved automatically by gaining the approval from Bahrain’s research committee.

## CONFLICT OF INTEREST

The authors have no conflicts of interest to declare.

## Figures and Tables

**Table 1.** Basic characteristics of the participants.

		**Count**	**%**
Sex	Male	471	53.4
	Female	411	46.6
Age group (years)	0–9	29	3.3
	10–19	110	12.5
	20–29	102	11.6
	30–39	143	16.2
	40–49	123	13.9
	50–59	167	18.9
	60–69	143	16.2
	70+	65	7.4
Age (mean ± SD)		42.8 ± 19.7	
Nationality	Bahraini	698	79.1
	Non-Bahraini	184	20.9
Diabetes mellitus (*N* = 877)	No	681	77.7
	Yes	196	22.3
Cancer (*N* = 877)	No	860	98.1
	Yes	17	1.9
Transplant (*N* = 877)	No	872	99.4
	Yes	5	0.6
Autoimmune (*N* = 876)	No	855	97.6
	Yes	21	2.4

**Table 2.** Clinical presentation and complications of herpes zoster.

		**Count**	**%**
Chief complaint	Itching	21	2.4
	Others	17	1.9
	Pain	139	15.8
	Rash	705	79.9
Location (*N* = 776)	Abdomen	108	13.9
	Back	151	19.5
	Face	100	12.9
	Lower limbs	86	11.1
	Multiple sites	7	0.9
	Trunk	237	30.5
	Upper limbs	87	11.2
Laterality (*N* = 658)	Right	356	54.1
	Left	294	44.7
	Both sides	8	1.2
Antiviral use (*N* = 684)	No	238	34.8
	Yes	446	65.2
Admission (*N* = 684)	No	675	98.7
	Yes	9	1.3
Complications (*N* = 684)	No	579	84.6
	Yes	105	15.4

**Table 3.** Rate of complications among patients who responded to phone calls (*N* = 684).

		**Count**	**%**
Cellulitis	No	654	95.6
	Yes	30	4.4
Post-herpetic neuralgia	No	638	93.3
	Yes	46	6.7
Ramsay Hunt – facial palsy	No	678	99.1
	Yes	6	0.9
Recurrence	No	671	98.1
	Yes	13	1.9
Others	No	645	96.4
	Yes	24	3.6

**Table 4.** Logistic regression analysis of factors associated with the presence of complications after herpes zoster infection.

	**OR**	***p***	**95% CI for OR**
**Sex**			
Male	Reference		
Female	0.81	0.340	0.52–1.26
**Nationality**			
Bahraini	Reference		0.66–2.10
Non-Bahraini	1.18	0.585	
**Presence of diabetes mellitus**			
No	Reference		
Yes	1.28	0.352	0.76–2.13
**Presence of cancer**			
No	Reference		
Yes	0.35	0.283	0.05–2.38
**Case of transplant**			
No	Reference		
Yes	7.07	0.072	0.84–59.67
**Presence of autoimmune diseases**			
No	Reference		
Yes	1.9	0.278	0.6–6.04
**Use of antivirals**			
No	Reference		
Yes	1.96	**0.008**	1.2–3.22
**Age 50 years and above**			
No	Reference		
Yes	2.82	**<0.001**	1.73–4.59

Bold values indicate being more than 50 and using antiviral treatment are associated with higher rates of HZ complications and it is statistically significant.

## References

[1] CDC Shingles (Herpes Zoster). https://www.cdc.gov/shingles/hcp/clinical-overview/index.html.

[2] Johnson RW, Bouhassira D, Kassianos G, Leplège A, Schmade KE, Weinke T (2010;). The impact of herpes zoster and post-herpetic neuralgia on quality of life. BMC Med.

[3] Kawai K, Gebremeskel BG, Acosta CJ (2014). Systematic review of incidence and complications of herpes zoster: Towards a global perspective. BMJ Open.

[4] John AR, Canaday DH (2017). Herpes Zoster in the older adult. Infect Dis Clin North Am.

[5] Cadogan SL, Mindell JS, Breuer J, Hayward A, Warren-Gash C (2022). Prevalence of and factors associated with herpes zoster in England: A cross-sectional analysis of the Health Survey for England. BMC Infect Dis.

[6] Opstelten W, Van Essen GA, Schellevis F, Verheij TJM, Moons KGM (2006). Gender as an independent risk factor for herpes zoster: A population-based prospective study. Ann Epidemiol.

[7] Yawn BP, Saddier P, Wollan PC, St. Sauver JL, Kurland MJ, Sy LS (2007). A population-based study of the incidence and complication rates of herpes zoster before zoster vaccine introduction. Mayo Clin Proc.

[8] Gauthier A, Breuer J, Carrington D, Martin M, Rémy V (2009). Epidemiology and cost of herpes zoster and post-herpetic neuralgia in the United Kingdom. Epidemiol Infect.

[9] Zoch-Lesniak B, Tolksdorf K, Siedler A (2018). Trends in herpes zoster epidemiology in Germany based on primary care sentinel surveillance data, 2005–2016. Hum Vaccin Immunother.

[10] Yin D, Van Oorschot D, Jiang N, Marijam A, Saha D, Wu Z (2021). A systematic literature review to assess the burden of herpes zoster disease in China. Expert Rev Anti Infect Ther.

[11] Stein AN, Britt H, Harrison C, Conway EL, Cunningham A, MacIntyre CR (2009). Herpes zoster burden of illness and health care resource utilisation in the Australian population aged 50 years and older. Vaccine.

[12] Weitzman D, Shavit O, Stein M, Cohen R, Chodick G, Shalev V (2013). A population based study of the epidemiology of Herpes zoster and its complications. J Infect.

[13] Soysal A, Gönüllü E, Yıldız İ, Karaböcüoğlu M (2021). Incidence of varicella and herpes zoster after inclusion of varicella vaccine in national immunization schedule in Turkey: Time trend study. Hum Vaccin Immunother.

[14] Al-Dahshan A, Chehab M, Ganesan N, Bansal D, Farag E, Al-Romaihi H (2020). Epidemiology of herpes zoster in the State of Qatar, 2012–2017. Qatar Med J.

[15] Alakloby OM, Aljabre SH, Randhawa MA, Alzahrani AJ, AlWunais KM, Bukhari IA (2008). Herpes zoster in eastern Saudi Arabia: Clinical presentation and management. J Drugs Dermatol.

[16] Harvey M, Prosser LA, Rose AM, Ortega-Sanchez IR, Harpaz R (2020). Aggregate health and economic burden of herpes zoster in the United States: illustrative example of a pain condition. Pain.

[17] Pan CX, Lee MS, Nambudiri VE (2022). Global herpes zoster incidence, burden of disease, and vaccine availability: A narrative review. Ther Adv Vaccines Immunother.

[18] Harpaz R, Ortega-Sanchez IR, Seward JF (2008). Prevention of Herpes Zoster recommendations of the Advisory Committee on Immunization Practices (ACIP). CDC.

[19] (2013). National Library of Medicine US. Shingles prevention study.

[20] Babamahmoodi F, Alikhani A, Ahangarkani F, Delavarian L, Barani H, Babamahmoodi A (2015). Clinical manifestations of herpes zoster, its comorbidities, and its complications in North of Iran from 2007 to 2013. Neurol Res Int.

[21] Esteban-Vasallo MD, Domínguez-Berjón MF, Gil-Prieto R, Astray-Mochales J, Gil De Miguel Á (2014;). Sociodemographic characteristics and chronic medical conditions as risk factors for herpes zoster: A population-based study from primary care in Madrid (Spain). Hum Vaccin Immunother.

[22] Kim YJ, Lee CN, Lim CY, Jeon WS, Park YM (2014). Population-based study of the epidemiology of herpes zoster in Korea. J Korean Med Sci.

[23] Esteban-Vasallo MD, Gil-Prieto R, Domínguez-Berjón MF, Astray-Mochales J, Gil de Miguel A (2014). Temporal trends in incidence rates of herpes zoster among patients treated in primary care centers in Madrid (Spain), 2005–2012. J Infect.

[24] Vukelić D, Končić DO, Prepolec J, Škrabić I, Parun AŠ, Skuhala T (2020). Clinical characteristics of hospitalized adults and adolescents with herpes zoster in Croatia: More than 20 years of a single-center experience. Croat Med J.

[25] Binsaeedu AS, Bajaber AO, Muqrad AG, Alendijani YA, Alkhenizan HA, Alsulaiman TA (2022). Clinical and epidemiological aspects of herpes zoster disease in a primary care setting in Riyadh, Saudi Arabia: A retrospective cohort study. J Family Med Prim Care.

